# The Role of *egr1* in Early Zebrafish Retinogenesis

**DOI:** 10.1371/journal.pone.0056108

**Published:** 2013-02-06

**Authors:** Liyun Zhang, Jin Cho, Devon Ptak, Yuk Fai Leung

**Affiliations:** 1 Department of Biological Sciences, Purdue University, West Lafayette, Indiana, United States of America; 2 Department of Biochemistry and Molecular Biology, Indiana University School of Medicine Lafayette, West Lafayette, Indiana, United States of America; Institut de la Vision, France

## Abstract

Proper retinal cell differentiation is essential for establishing a functional retina. The purpose of this study is to investigate the role of *early growth response 1* (*egr1*), a transcription factor (TF) that has been reported to control eye development and function, on retinal differentiation in zebrafish. Specifically, cellular changes in the Egr1-knockdown retinas were characterized by immunohistochemistry at 72 and 120 hours post-fertilization (hpf). The results indicate that Egr1 knockdown specifically suppressed the differentiation of subtypes of amacrine cells (ACs) and horizontal cells (HCs), including Parvalbumin- and GABA-positive ACs as well as Islet1-positive HCs. In addition, the knockdown induced a general delay of development of the other retinal cell types. These differentiation problems, particularly the ones with the ACs and HCs, also compromised the integrity of the inner and outer plexiform layers. In the Egr1-knockdown retinas, the expression of *ptf1a,* a TF that controls the specification of ACs and HCs, was prolonged and found in ectopic locations in the retina up to 72 hpf. Then, it became restricted to the proliferative marginal zone as in the control retinas at 120 hpf. This abnormal and prolonged expression of *ptf1a* during retinogenesis might affect the differentiation of ACs and HCs in the Egr1-knockdown retinas.

## Introduction

The vertebrate retina is consisted of six types of neurons and one major type of glial cell [1]. These cells are organized into a laminated structure characterized by three distinctive cellular layers including the ganglion cell layer (GCL), inner nuclear layer (INL) and outer nuclear layer (ONL). These cellular layers are separated by two synaptic layers including the inner plexiform layer (IPL) and outer plexiform layer (OPL). The GCL is consisted mainly of ganglion cells (GCs) and a low number of displaced amacrine cells (DACs) which are located next to the IPL. The INL is consisted of three types of neurons: ACs, bipolar cells (BCs) and HCs, which are distributed in the inner, middle and outer part of the INL respectively. The cell body of Müller cells (MCs), the major glial cell type, is also located in the middle part of INL. The ONL is composed of the cell bodies of both rod and cone photoreceptors (PRs).

During the course of retinal development in vertebrates, the retinal progenitor cells are capable to produce all types of retinal cells in a conserved order. Generally, GCs are the first cell type to be generated. This is followed by overlapping births of the other cell types with MCs being the last type to be formed [2]. Ultimately, these retinal cells terminally differentiate, synapse with each other and establish a laminated structure. A number of signal transduction pathways and processes have been shown to regulate retinal lamination through studies in zebrafish, mouse and chick. These included *sonic hedgehog a* (*shha*) [3], cell adhesion [4], [5], [6], [7], [8], cell polarity regulation [9] and chromatin remodeling [10]. For example, our group characterized the zebrafish mutant of *smarca4,* which encodes the ATPase of SWI/SNF chromatin remodeling complex [11], by gene expression analysis. A number of genes were found to be differentially expressed in the mutant dystrophic retinas [12], [13]. One of these downstream targets, *irx7*, was subsequently demonstrated to be essential for retinal differentiation [14]; therefore, further characterization of these *smarca4*-regulated genes will provide new insights into the mechanistic details of retinal development.

*Early growth response 1* (*egr1*), a zinc finger TF, is another *smarca4*-regulated gene. It was originally identified as an early response gene that rapidly responded to different growth stimuli [15] and involved in cell proliferation [16], differentiation [17] as well as synaptic plasticity [18]. The function of *egr1* is also diverse in retina. For example, it has been shown that *egr1* was activated by MAPK during MCs proliferation and trans-differentiation into progenitors in acutely-damaged chicken retina [19]. Also, *egr1* expression was differentially regulated in chick retina according to the sign of defocus lens applied to the animals [20], reduced in form-deprived eyes in mice [21] and reduced in both hyperopically- and myopically-defocused eyes in monkeys [22]. Besides, *Egr1*-null mice was myopic [23]. In zebrafish retina, *egr1* was expressed at an early stage of development between 40–48 hpf [24]. In addition, Egr1 knockdown in zebrafish led to a smaller eye with defects in retinal differentiation and lamination [25]. Coincidently, our ongoing *in situ* hybridization study has shown that *egr1* is suppressed in the *smarca4*-mutant retinas (unpublished data), suggesting that *egr1* is a downstream effector of the *smarca4*-regulated gene network. However, it is not clear how the attenuation of *egr1* expression would result in defects in retinal development.

The current study has further defined the roles of *egr1* in retinal development by morpholino (MO) knockdown experiments. At the early stage of retinogenesis, a normal Egr1 expression was essential for proper differentiation of cells in the INL and ONL, as well as the neurite outgrowth of GCs. In older embryos, different cell types in the INL and ONL differentiated better and became comparable to the controls, except for Parvalbumin+ and GABA+ ACs, as well as Islet1+ HCs. These findings indicate that there was a specific defect in the differentiation of AC and HC subtypes in the Egr1-knockdown retinas; while for the other retinal cell types, the knockdown caused a delay in their differentiation.

## Results

### The expression dynamics of *egr1* during retinogenesis

**Figure 1:**
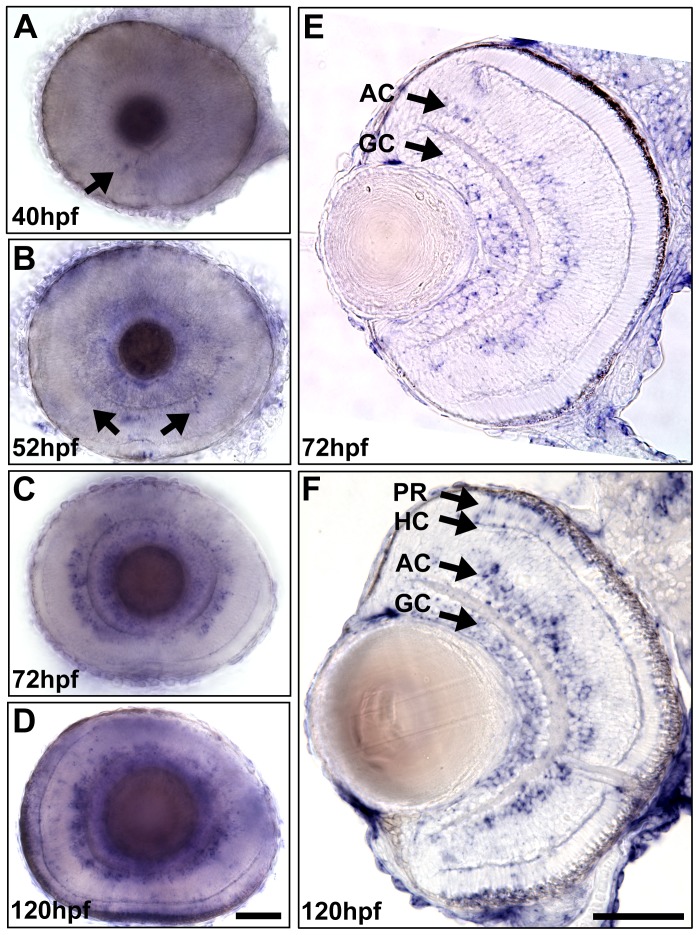
The expression dynamics of *egr1* during zebrafish retinogenesis. A time-series whole-mount *in situ* hybridization was performed to detect the expression pattern of *egr1* in the WT retina. The signal of *egr1* was first detected in the anterior-ventral retina at 40 hpf (A, arrow). Then, *egr1* expression spread to the dorsal retina at 52 hpf (B, arrows). At 72 hpf (C & E), strong signal was detected in the AC and GC regions. Occasionally, positive staining was observed in the HC and PR regions, but it did not become prominent in the peripheral outer retina until 120 hpf (D & F). At this stage, the signal was relatively intense in the GCL and AC region. GC: ganglion cells; AC: amacrine cells; HC: horizontal cells; PR: photoreceptors. Scale bars  =  50 µm.

To obtain an expression pattern of *egr1* during retinal development, *in situ* hybridization was conducted on wild-type (WT) embryos at 24, 28, 36, 40, 44, 48, 52, 60, 72, 84, 96 and 120 hpf (Figure 1 and data not shown). The first detectable signal of *egr1* was found in the anterior-ventral retina at 40 hpf (Figure 1A). This is the approximate stage when the INL cells in the same region begin to withdraw from cell cycle [26]. Contrary to a previous report that *egr1* expression in retina was detected at 40 hpf and disappeared by 48 hpf [24], the results in this study show that *egr1* continued to express and spread to the dorsal retina. The signal was detected in the GCL in addition to the AC region at 52 hpf (Figure 1B), a stage when the retinal lamination is first established. The staining signal of *egr1* became more intense by 72 hpf, and was mainly detected in the GCL and AC regions (Figure 1C & E). The late retinal expression of *egr1* is further supported by the *in situ* hybridization data at zfin.org that show an intense retinal staining at the protruding-mouth stage (∼72 hpf) (ZDB-GENE-980526-320). In addition, a few cells in the HC and PR regions also began to express *egr1* starting at this stage. The signal in these regions became more prominent, particularly in the peripheral outer retina, by 120 hpf (Figure 1D & F). The initial expression dynamics of *egr1*, which was primarily located in the proximity of the IPL during its first establishment, hints at the possibility of its role in guiding the differentiation of cells in this area. The later appearance of *egr1* in PRs suggests that it may play a role in PR differentiation and/or function, a role that has been implicated by other investigations [27].

### Egr1 knockdown reduced eye size and compromised retinal lamination at early stage of retinogenesis

**Figure 2:**
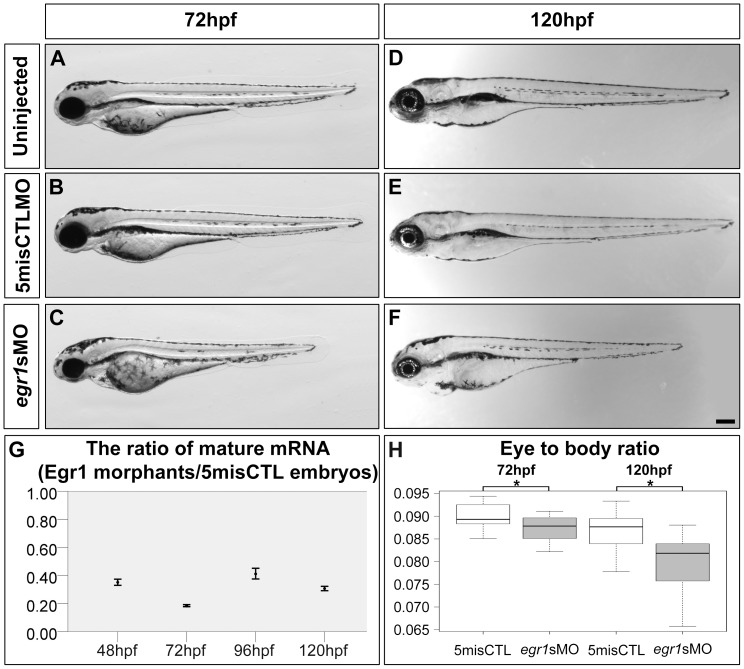
Egr1 was substantially knocked down by antisense morpholino up to 120 hpf. Compared with the uninjected embryos (uninjected; A) and controls (5misCTLMO; B) which had no obvious change in gross morphology, 89.3% (134/150) of the Egr1 morphants (*egr1s*MO; C) displayed a reduced eye size and a shortened body trunk at 72 hpf. This problem persisted to 120 hpf, at which the Egr1 morphants (F) were still noticeably shorter than the controls (D & E). Scale bar  =  200 µm. The amount of the mature *egr1* mRNA in the samples was measured by qPCR in order to determine the efficiency of the splice-blocking effect. The ratio and ratio range of the mRNA between Egr1 morphants and controls at 48, 72, 96 and 120 hpf were plotted in (G). The calculation of ratio and ratio range was conducted with the standard ΔΔCt method [47] as described in Methods. Overall, only about 20–40% of mature mRNA was detected in the Egr1 morphants until 5 dpf. (H) A boxplot of the eye-to-body ratios of the controls and Egr1 morphants. The anterior-posterior length of the eyes, when normalized by the body length, revealed a specific reduction of the eye size in the Egr1 morphants at both 72 and 120 hpf. Asterisks: *p*<0.05.

To determine *egr1*’s function in retinal development, Egr1 was knocked down by microinjection of MOs in developing zebrafish embryos and the resultant retinal phenotypes examined. A splice-blocking MO (*egr1*sMO) (Figure 2) and a translation-blocking MO (*egr1*tMO) were used. Since the retinal phenotypes of these two types of MO-knockdown embryos (morphants) were comparable, the results obtained by the injection of 4 ng of *egr1*sMO are presented below. This amount was chosen because of the following two reasons: first, the gross morphology of the embryos injected with 4 ng of 5-base mismatch control MO (5misCTL MO; the embryos injected with this MO will be referred to as controls hereafter) (Figure 2B) was indistinguishable from the uninjected embryos (Figure 2A) at 72 hpf; while the injection of the same amount of *egr1*sMO led to a reduction of eye and head size, as well as a general shortening of the trunk in most of the morphants (Figure 2C) (N = 134 out of 150, or 89.3%). The remaining morphants (N = 16 out of 150, or 10.7%) were unhealthy and excluded from subsequent characterizations. This morphological problem caused by Egr1 knockdown persisted to 120 hpf (Figure 2F), when the uninjected embryos (Figure 2D) and controls (Figure 2E) were still highly comparable to each other and healthy. Second, there was a substantial reduction of the amount of *egr1* mRNA in the Egr1 morphants to 20–40% of the control level up to 120 hpf, as determined by quantitative PCR (qPCR) (Figure 2G). This suggests that Egr1 expression was substantially reduced in the morphants. Contrary to a previous report [25], Egr1 knockdown in this study did not induce a wide range of phenotypes. Since the injection volume in this study was calibrated and the resulting injected embryos were screened for the evenness of the fluorescence signal from the FITC-dextran tracer, it is believed that the phenotypes that were observed in this study are genuine.

To quantify the effect of Egr1 knockdown on eye size, the anterior-posterior length of the eyes was measured as described [28]. The results indicate that there was a reduction of eye size in the Egr1 morphants (mean (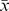) = 252.60 µm, standard deviation (*s*) = 14.37 µm, N = 19) compared with the controls (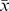 = 301.36 µm, *s* = 7.05 µm, N = 10) (Mann-Whitney test, U = 0, *p*-value<0.001) at 72 hpf. After normalizing the eye length with the body length of the same embryo, the morphants still had a smaller eye/body ratio compared with the controls (Figure 2H; morphants: 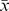 = 0.0866, *s = *0.0042; controls: 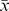 = 0.0899, *s  = * 0.0031; Mann-Whitney test, U = 49, *p*-value = 0.035). This reduction in eye/body ratio persisted to 120 hpf (Egr1 morphants: 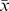 = 0.0794, *s = *0.0066, N = 21; controls: 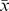 = 0.0874, *s = *0.0029, N = 16; Mann-Whitney test, U = 44, *p*-value<0.001). Together with a substantial reduction of *egr1* mRNA at this stage, these results indicate that while there was a general reduction in the body size of the Egr1 morphants, there was also a specific reduction of the eye size in these embryos.

**Figure 3:**
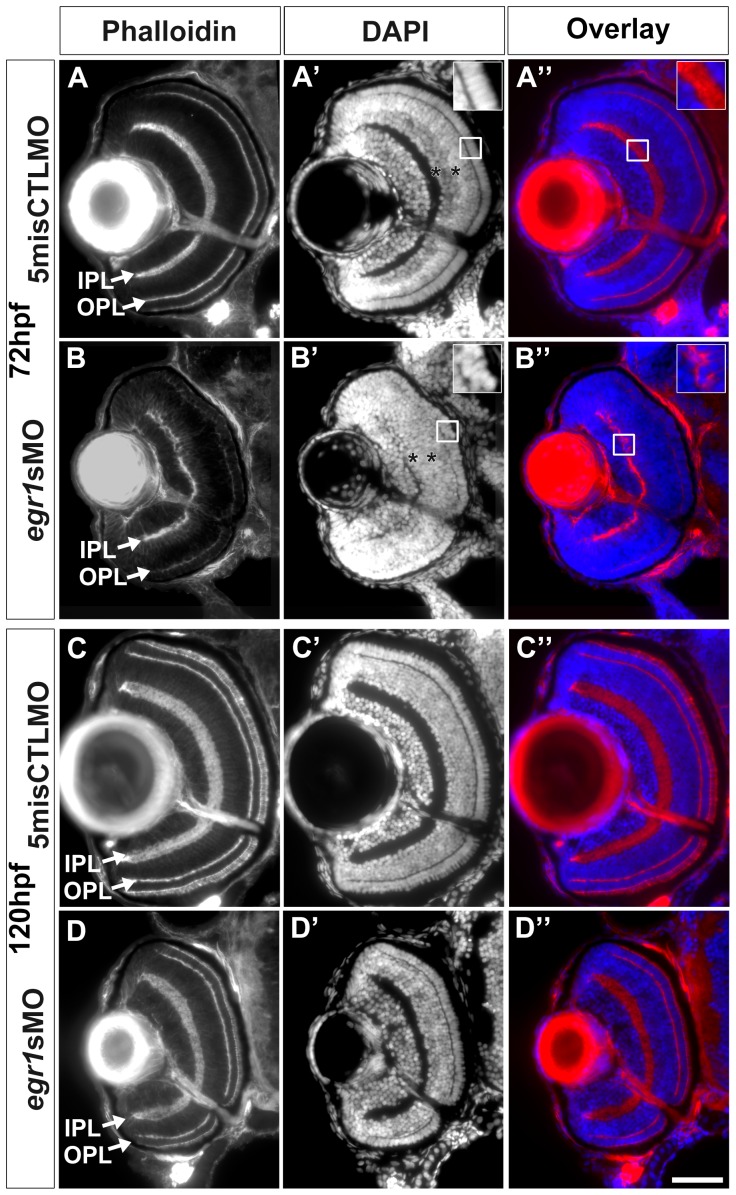
Egr1 knockdown compromised retinal differentiation and lamination at 72 hpf, and these defects were mostly resolved by 120 hpf. The retinal histology of the Egr1-morphant retinas was analyzed by immunofluorescence. (A & B) The IPL and OPL (arrows) that were stained with phalloidin were thinner and irregular in the Egr1 morphants (*egr1s*MO) compared with the controls (5misCTLMO) at 72 hpf. A similar observation of the plexiform layer formation was also made with the DAPI nuclei stain on the same sections (A’ and B’). The DAPI stain also revealed issues in the differentiation of INL and ONL. For example, while a more intense apical sub-layer and a less intense basal sub-layer were observed in the INL of the controls (A’, asterisks), this distinction was not apparent in the Egr1 morphants (B’, asterisks). This suggests that the differentiation of the INL was compromised in the morphant retinas. Further, the nuclei in the ONL of the Egr1 morphants (B’, inset) were less elongated than that in the controls (A’, inset), suggesting that PR differentiation was affected by Egr1 knockdown. The overlay pictures of phalloidin and DAPI also demonstrate that the irregularity of the IPL was caused by mis-placed cells in the IPL in the Egr1 morphants (B”, inset); while no mis-placed cells were found in the controls (A”, inset). By 120 hpf, many of these differentiation problems in the Egr1-morphant retinas were largely resolved. For example, the IPL and OPL were more regular and their phalloidin staining was more intense (D) and was comparable to the controls (C). Nonetheless, the IPL was still thinner in the Egr1 morphants. In addition, the PRs in the Egr1 morphants was not stained as intensely by phalloidin as the controls, even though the differentiation of PRs became relatively normal at this stage (see Figure 7). For all sections, the lens is on the left and dorsal is up. IPL: inner plexiform layer; OPL: outer plexiform layer. Scale bar  =  50 µm.

The retinal structure of the Egr1 morphants was abnormal compared with the controls at 72 hpf, a stage at which the retinas are mature enough to elicit visual activity [26]. First, the retinal lamination was not formed properly. The IPL and OPL that were highlighted by phalloidin were thinner and irregular in the Egr1 morphants compared with the controls (Figure 3A & B, arrows). The irregularity of the IPL was even more apparent in the sections stained by DAPI which highlighted the nuclei (Figure 3A’ & B’). In addition, some nuclei were mis-placed in the IPL and surrounded by the phalloidin signal (Figure 3A’’ & B’’, insets). Second, the nuclei of the INL cells were not stained as an intense apical sub-layer and a less intense basal sub-layer (Figure 3A’ & B’, asterisks). Moreover, cells in the ONL were less elongated (Figure 3B’, inset) compared with controls (Figure 3A’, inset), suggesting that PR differentiation might also be affected by Egr1 knockdown at this stage. The retinal lamination problem in the Egr1 morphants was largely resolved by 120 hpf (Figure 3D). In particular, the IPL and OPL in the Egr1 morphants were relatively normal compared with their counterparts at 72 hpf (Figure 3B) and were more comparable to the controls (Figure 3C). Nonetheless, the IPL remained thinner at this stage, as supported by measurements of the IPL thickness in the central retina (Egr1 morphants: 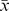 = 12.39 µm, *s = *2.13 µm, N = 50; controls: 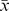 = 13.62 µm, *s  = * 2.51 µm, N = 33; two-tailed Student’s *t*-test, *p*-value  =  0.019) Consistent with the morphometric analysis, the overall size of the Egr1-morphant retinas was still smaller at 120 hpf. In addition, the phalloidin staining of the outer segments of PRs in the Egr1 morphants was still less intense compared with the controls (Figure 3C & D).

### Egr1 knockdown specifically affected the differentiation of AC subtypes

**Figure 4:**
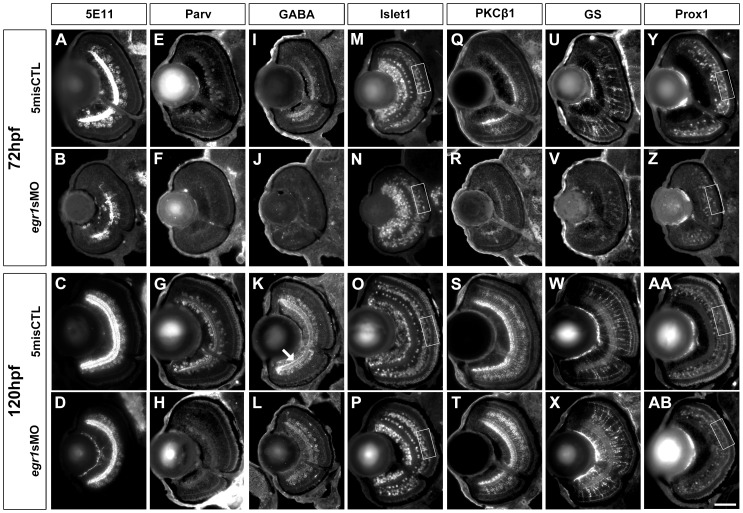
Immunohistochemical analysis of the INL cells in the Egr1-morphant retinas. Immunohistochemical analysis of the INL cells in the controls (5misCTLMO) and Egr1 morphants (*egr1s*MO) was performed with several cell markers at 72 and 120 hpf. These include anti-5E11 (5E11; A-D), anti-parvalbumin (Parv; E-H), anti-GABA (GABA; I-L) and anti-Islet1 (Islet1; M-P) for ACs; anti-PKCβ1 (PKC; Q-T) for BCs; anti-GS (GS; U-X) for MCs; and Islet1 and anti-Prox1 (Prox1; Y-AB) for HCs. In short, the analysis has revealed that Egr1 knockdown specifically compromised the differentiation of Parv+ and GABA+ ACs. See text, Table 1 and 2 for further discussion and additional results for the specific effects on HCs differentiation in Figure 6. For all sections, the lens is on the left and dorsal is up. Scale bar  =  50 µm.

To further define the effect of Egr1 knockdown on retinal differentiation, immunostaining analysis of cells located in the INL was conducted with embryos collected at 72 and 120 hpf. Since *egr1* begins to express in the AC region during retinogenesis (Figure 1), the analysis was first focused on ACs, an early retinal cell type that would be generated in this region. The markers used in the analysis include (1) anti-5E11 (5E11; Figure 4A–D), a pan-specific AC marker [29]; (2) anti-Parvalbumin (Parv; Figure 4E–H) [30] and (3) anti-GABA (GABA; Figure 4I–L) for GABAergic ACs [31]. The Parv marker labels a subset of GABA+ ACs (Figure S1); and (4) anti-Islet1 (Islet1; Figure 4M–P) for ACs which were shown to be cholinergic in mice [32]. This marker was also used to label a subset of ACs in zebrafish [33]. The results are also summarized in Table 1. The signal of 5E11 was substantially and moderately reduced in the Egr1-morphant retinas at 72 and 120 hpf respectively (Figure 4B & D); while the corresponding controls had extensive signal in the ACs and their projections into the IPL (Figure 4A & C). These observations suggest that ACs differentiation was compromised by Egr1 knockdown. This differentiation problem was also revealed by the analysis of three additional markers of AC subtypes. First, Parv+ ACs were mostly absent in the Egr1 morphants compared with the controls at both 72 and 120 hpf (Figure 4E–H). Second, GABA+ ACs were mostly absent in the Egr1 morphants at 72 hpf compared with the controls (Figure 4I & J); while the staining for these cells became more apparent by 120 hpf (Figure 4L), except for the intense staining on the basal IPL that was only observed in the controls (Figure 4K, arrow). Since the GABA staining in the normal retina overlapped substantially with Parv (Figure S1), this suggests that the suppressed GABA+ ACs might also be Parv+. Third, the number of Islet1+ ACs per retinal area was not reduced at both 72 hpf (Figure 4M & N and Table 2; Mann-Whitney Test, *p*-value  =  0.641) and 120 hpf (Figure 4O & P and Table 2; Mann-Whitney Test, *p*-value  =  0.705). Together, these results suggest that Egr1 knockdown specifically affected the differentiation of Parv+ and GABA+ ACs.

**Table 1:** A summary of the immunostaining analysis of cell-type specific makers in the Egr1-morphant retinas.

Cell type	Cell Maker	At 72 hpf	At 120 hpf	Figure
GC	zn8	(-), reduced dendritic projection	(-)	5
AC	5E11	↓	↓	4A-D
	Parv	↓↓	↓↓	4E-H
	GABA	↓↓	↓	4I-L
	Islet1	(-)	(-)	4M-P
HC	Prox1 and DAPI	↓	(-)	4Y-AB; 6A-D
	Islet1	↓	↓	6E-H
BC	PKCβI	↓↓	(-)	4Q-T
MC	GS	↓	(-)	4U-X
Cone	zpr1	↓↓	(-)	7A-B, E-F
Rod	zpr3	↓↓	(-)	7C-D, G-H

(-): no obvious change between the Egr1 morphants and controls

↓: intermediate reduction compared with the controls

↓↓: severe reduction compared with the controls

The immunostaining analysis results are summarized according to their cell type and markers used. The extent of the staining at 72 and 120 hpf is presented by the following scheme: (-): no obvious change between the Egr1 morphants and controls; ↓: intermediate reduction compared with the controls; ↓↓: severe reduction compared with the controls. The figure numbers of the corresponding immunostaining pictures are also listed.

**Table 2:** A statistical summary of the cell marker staining results.

		5misCTL			*egr1*sMO					
Cells	Hours post fertiliztion (hpf)	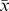 (mm^-2^)	*s* (mm^-2^)	N	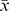 (mm^-2^)	*s* (mm^-2^)	N	Mann-Whitney test U value	*P* value	Figure
Islet1+ ACs	72	1335	134	8	1377	177	17	60	0.641	4M & N
	120	1273	161	10	1311	145	10	45	0.705	4O & P
Zn8+ GCs	72	7530	727	9	7961	819	18	62	0.328	5A & B
Prox1+ HCs	72	1368	137	11	749	190	7	1	< 0.001	6A & B
	120	2079	278	10	2140	182	20	93	0.779	6C & D
Islet1+ HCs	72	1304	193	8	784	120	17	0	< 0.001	6E & F
	120	1115	118	10	681	270	9	8	0.003	6G & H

For Islet1+ACs, zn8+ GCs, and Prox1+ & Islet1+ HCs, their numbers were counted and normalized by the corresponding retinal area. The mean (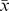), standard deviation (*s*) and the number of embryos (N) for each group at each stage are listed, and the corresponding U- and *p*-values from the Mann-Whitney test computed. The figure numbers of the corresponding immunostaining pictures are also listed.

To determine the extent to which the differentiation problem of ACs was caused by a delay in development, immunostaining analysis of BCs and MCs, two late cell types in retinogenesis, was conducted. The markers used in this analysis include anti-PKCβ1 for BCs (PKCβ1; Figure 4Q–T) and anti-GS for MCs (GS; Figure 4U–X). While the staining of both PKCβ1+ BCs and GS+ MCs was suppressed in the Egr1 morphants (Figure 4R & V) compared with the controls (Figure 4Q & U) at 72 hpf, the expression of these markers was very comparable between the two groups at 120 hpf (Figure 4S & T; W & X). The suppression of MCs at 72 hpf but not at 120 hpf indicates that the differentiation defects at 72 hpf, including the malformation of the IPL (Figure 3B), were most likely caused by a delay in development induced by Egr1 knockdown. Nonetheless, the persistent of the suppression of Parv+ ACs (Figure 4H) and attenuation of GABA+ ACs (Figure 4L) amidst the recovery of MCs (Figure 4X) in the Egr1 morphants at 120 hpf strongly suggests that the suppression of these AC subtypes was a specific effect of Egr1 knockdown.

### Egr1 knockdown compromised the normal dendritic projection of GCs into the IPL

**Figure 5:**
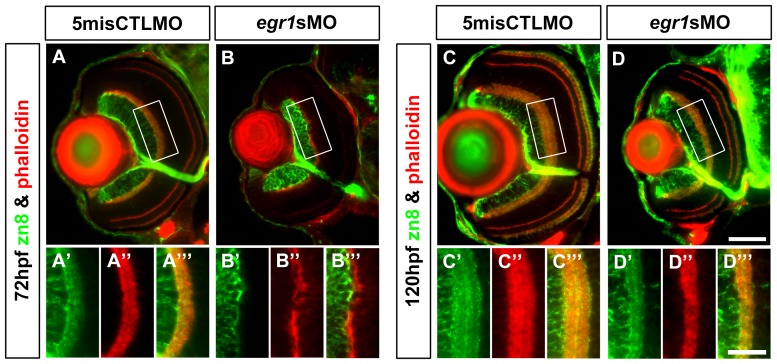
Immunohistochemical analysis of the GCs in the Egr1-morphant retinas. Immunohistochemical analysis of the GCs in the controls (5misCTLMO) and Egr1 morphants (*egr1s*MO) was performed by anti-zn8 (zn8; green) at 72 hpf (A & B) and 120 hpf (C & D). Phalloidin (red) was used as a counterstain to highlight the plexiform layers. A whole-eye section is shown at the top for each condition, while the magnified view of a selected region (white box) on the dorsal side of the optic nerve is shown at the bottom. The analysis has indicated that Egr1 knockdown suppressed the early dendritic outgrowth of GCs into the IPL at 72hpf (B), which was irregular at this stage. In addition, the cell number per retinal area was not different between the two groups. This defect was largely resolved by 120 hpf, despite the IPL was still thinner as shown in Figure 3. This suggests that there were still defects in differentiation of cells that projected neurites into the IPL. One possible cause of the defect is the differentiation problem of ACs as shown in Figure 4. See text, Table 1 and 2 for further discussion. For the whole-eye sections, the lens is on the left and dorsal is up. Scale bar  =  50 µm for the whole-eye sections and 25 µm for the selected regions.

The lack of presynaptic projections from the INL cells would probably affect the differentiation of their postsynaptic partners in the GCL. In the meantime, *egr1* also begins to express in the GCL at 52 hpf (Figure 1); hence, it was theorized that the differentiation of GCL would be affected by Egr1 knockdown. To investigate this possibility, immunostaining analysis of GCs and their dendritic projections into the IPL was conducted with anti-zn8 (zn8) at 72 and 120 hpf (Figure 5). The results are also summarized in Table 1. At 72 hpf, the differentiation of zn8+ GCs was compromised in the Egr1-morphant retinas. Specifically, the dendritic projection of the GCs into the IPL was almost absent (Figure 5B) when compared with the controls (Figure 5A). Intriguingly, the number of zn8+ GCs per retinal area was not different between the two groups (Table 2; Mann-Whitney Test, *p*-value  =  0.328), suggesting that the dendritic outgrowth of the zn8+ GCs was preferentially affected compared with the soma at this stage. By 120 hpf, the GCs in the Egr1-morphant retinas could extend dendritic projections into the IPL (Figure 5D), which was still thinner than the controls (Figure 5C). This is also supported by the IPL-thickness measurements as described above. Together, these observations indicate that the dendritic differentiation of GCs was substantially delayed in the Egr1 morphants. Nonetheless, the current experimental design did not discriminate whether this delay was a direct effect of Egr1 knockdown or a secondary effect induced by the differentiation defects of AC subtypes.

### Defects in the outer retina of the Egr1 morphants

**Figure 6:**
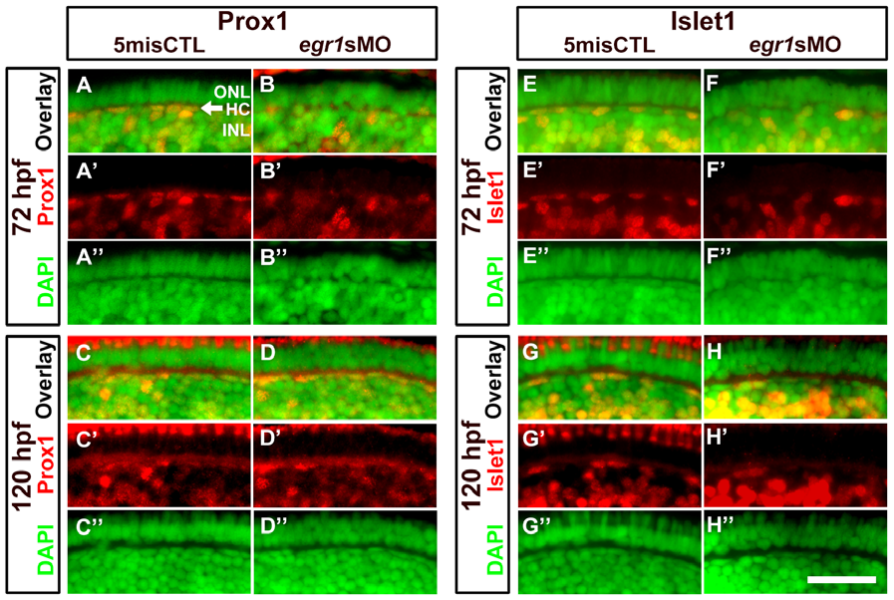
Immunohistochemical analysis of the HCs in the Egr1-morphant retinas. A magnified view of the immunostaining results of HCs in the controls (5misCTLMO) and Egr1 morphants (*egr1s*MO) with Prox1 (A-D) and Islet1 (E-H) at 72 and 120 hpf. These selected regions correspond to the white boxes as shown in Figure 4. Prox1+ and Islet1+ cells are shown in red, while the DAPI nuclei counterstain is shown in green. The location of HCs is indicated by an arrow in A. See text, Table 1 and 2 for further discussion. In all pictures, the apical retina is to the top and dorsal is to the left. HC: horizontal cells; INL: inner nuclear layer; ONL: outer nuclear layer. Scale bar  =  50 µm.

The formation of the outer retina was also affected in the Egr1 morphants, particularly at the earlier stage 72 hpf. The defects could be caused by an abnormal differentiation of HCs and PRs that are in proximity of the OPL, as well as the BCs and MCs as described above. Since it has been reported recently that the reduction in HC number is related to OPL formation [14], a similar analysis was conducted using Islet1 (Figure 4M–P; a magnified view is shown in Figure 6E–H) and anti-Prox1 (Prox1; Figure 4Y-AB; a magnified view is shown in Figure 6A–D) to investigate the extent to which HCs were reduced in the Egr1 morphants. The results are also summarized in Table 1. The number of Prox1+ HCs per retinal area in the Egr1 morphants was reduced at 72 hpf compared with the controls (Figure 6A & B) (Table 2; Mann-Whitney Test, *p*-value < 0.001). At 120 hpf, since the Prox1 staining became relatively faint (Figure 6C & D), a phenomenon that was also observed in another study [31], the morphologically distinct HCs with flattened nuclei and detectable Prox1 signal were counted and normalized by the retinal area. The results indicate that the number of Prox1+ HCs per unit retinal area was not different between the two groups at this stage (Table 2; Mann-Whitney Test, *p*-value  =  0.779). Interestingly, there were fewer Islet1+ HCs per retinal area in the Egr1 morphants compared with the controls at both 72 hpf (Figure 6E & F and Table 2; Mann-Whitney Test, *p*-value < 0.001) and 120 hpf (Figure 6G & H and Table 2; Mann-Whitney Test, *p*-value  =  0.003). Thus, these results indicate that at least the differentiation of Islet1+ HCs was specifically affected by Egr1 knockdown.

**Figure 7:**
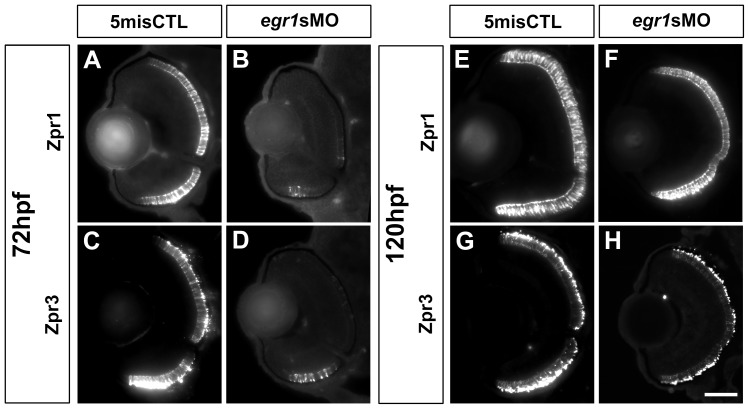
PR differentiation was delayed in the Egr1-morphant retinas. Immunohistochemical analysis of the PRs in the controls (5misCTLMO) and Egr1 morphants (*egr1s*MO) was performed with zpr1 (red-green double cones) and zpr3 (rods) at 72 hpf (A-D) and 120 (E-H) hpf. The signal of zpr1+ and zpr3+ cells was detected in the whole ONL of the controls at 72 hpf (A & C), while they were substantially reduced and restricted to a small region on the ventral ONL in the Egr1 morphants (B & D). Four staining types were defined as follows: Type 1: ≤ ¼, 2: ≤ ½, 3: ≤ ¾, 4  =  full retina. In these example images, the controls are staining Type 4 while the morphant images are staining Type 1. By 120 hpf, the differentiation of the zpr1+ and zpr3+ cells in the Egr1-morphant retinas (F & H) became more comparable to the controls (E & G). For all sections, the lens is on the left and dorsal is up. Scale bar  =  50 µm.

PR differentiation was investigated by immunostaining with anti-zpr1 (zpr1) for red-green double cones and anti-zpr3 (zpr3) for rods at 72 and 120 hpf. In the controls, zpr1+ and zpr3+ cells were detected in the whole ONL (Figure 7A & C) at 72 hpf, while these cells were primarily restricted to a small ventral region of the ONL in the Egr1 morphants at the same stage (Figure 7B & D). The results were then quantified by counting the number of sections with signal spanning from the ventral to a certain level of dorsal retina [14] (type 1: ≤ ¼, 2: ≤ ½, 3: ≤ ¾, 4  =  full retina). The results show that there was a difference in the counts between the controls and Egr1 morphants for zpr1 staining (control counts (type 1-4): 0, 0, 0, 14; Egr1-morphant counts: 10, 7, 10, 1; Mann-Whitney Test: U  =  7, *p*-value < 0.001) and zpr3 staining (control counts: 0, 0, 3, 13; Egr1-morphant counts: 20, 4, 2, 2; Mann-Whitney Test: U  =  22, *p*-value < 0.001). There was also a concomitant change in the *opsin* expression in different PR subtypes, including three *opsins* (*opn1lw1*: *red*, *opn1sw2*: *blue* and *opn1sw1*: *uv*) for three types of cone PRs and *rhodopsin* (*rho*) for rods (Figure S2). In addition, the expression of *nr2e3, neurod* and *crx,* three TFs that can specify PRs, was also investigated. The results show that the expression of *nr2e3* was increased and more widespread in the Egr1 morphants (Figure S3B) compared with the controls (Figure S3A). For *neurod* and *crx*, their expression between the controls and Egr1 morphants was similar (Figure S3C-F). Nonetheless, the signal of the zpr1+ and zpr3+ PRs in the Egr1 morphants became much more comparable to the controls by 120 hpf (Figure 7E–H). Taken together, these experiments suggest that Egr1 knockdown altered the differentiation of all types of PRs at 72 hpf, but the differentiation of PRs was more comparable to the controls by 120 hpf. Thus, Egr1 knockdown delayed PRs differentiation.

### Egr1 regulated the expression of *ptf1a* that specifies ACs and HCs

**Figure 8:**
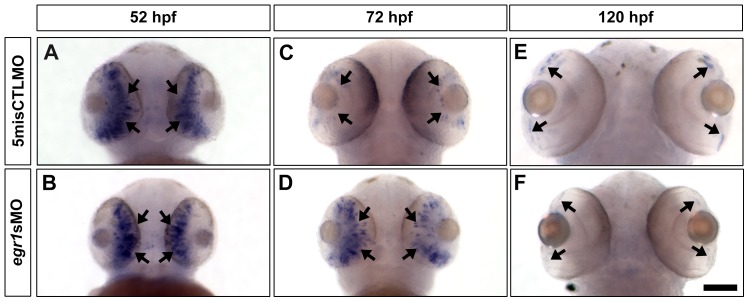
The expression of *ptf1a,* a TF that specifies ACs and HCs, was abnormal in the Egr1-morphant retinas. Whole-mount *in situ* hybridization of *ptf1a* was performed with the controls (5misCTLMO) and Egr1 morphants (*egr1s*MO) collected at 52, 72 and 120 hpf. At 52 hpf, *ptf1a* was primarily expressed in the differentiating retinal neuroepithelium (A & B, arrows) in both types of samples. By 72 hpf, the expression of *ptf1a* was restricted to the proliferative MZ in the controls (C, arrows), while its expression was maintained in the developing central retina in the Egr1 morphants (D, arrows). This ectopic expression was transient, as *ptf1a* was finally expressed in MZ in the Egr1 morphants (F, arrows) in a very comparable manner as the controls (E, arrows). The ventral view of the embryos is shown in all pictures. Scale bar  =  100 µm.

Since *egr1* is a TF, it is possible that it exerted its effect on the differentiation of ACs and HCs in the Egr1 morphants through transcriptional regulation of TFs that specify these cell types. To test this hypothesis, the expression of *ptf1a* that is transiently activated in all ACs and HCs precursors [31], [34] was studied in embryos collected at 52, 72 and 120 hpf by *in situ* hybridization (Figure 8). At 52 hpf, *ptf1a* was widely expressed in the developing neural retinas in both controls and Egr1 morphants (Figure 8A & B). By 72 hpf, the expression of *ptf1a* was restricted to the proliferative marginal zone (MZ) in the controls (Figure 8C), while there was still a noticeable ectopic expression in the INL of the Egr1 morphants (Figure 8D). The difference in the *ptf1a* expression pattern between the two groups diminished by 120 hpf, and the staining signal was detected in the MZs in both groups (Figure 8E–F).

## Discussion

Egr1 has been shown to play an important role in zebrafish retinal development in an earlier study [25]. In particular, the retinal structure of the knockdown embryos was disrupted and the retinal cells were immature. The signal of zpr1 and glutamate receptor 1 was substantially suppressed, indicating that Egr1 knockdown compromised differentiation of PRs, GCs and ACs. The current study has found similarities and differences in the cellular differentiation in the Egr1-morphant retinas. For example, for most immunostaining markers, even though there was a substantial reduction in their signals in the Egr1-morphant retinas at 72 hpf, the difference became diminished by 120 hpf (Table 1). Thus, our comprehensive marker analysis indicates that the differentiation problem of many cell types in the Egr1 morphants was likely caused by a developmental delay, except for Parv+ and GABA+ ACs as well as Islet1+ HCs. The differentiation of these cell types was still compromised in the Egr1-morphant retinas at 120 hpf (Figure 4 & Figure 6). Since the differentiation of MCs, the last cell type to be formed in retinogenesis, was comparable between the Egr1 morphants and controls at this stage; these results strongly suggest that the suppression of these AC and HC subtypes was a specific outcome of Egr1 knockdown.

The development of ACs is controlled by several TFs. For example, *islet1* is essential for cholinergic AC differentiation [32] while *neurod* has been shown to promote AC fate in mouse [35]. In addition, *ptf1a* is transiently expressed in the AC precursors in zebrafish [31]. In this study, Egr1 knockdown did not suppress Islet1+ ACs (Figure 4M–P) or alter *neurod* expression at 72 hpf (Figure 8E and F). These observations suggest that *egr1*’s effect on AC development was likely not mediated through *islet1* or *neurod*. However, there was a substantial and moderate suppression of Parv+ (Figure 4E–H) and GABA+ (Figure 4I–L) ACs in the Egr1 morphants respectively. Since most Parv+ ACs were also GABA+ (Figure S1), these results strongly indicate that *egr1* promotes the differentiation of Parv+ GABAergic ACs. For HCs, the immunostaining with Prox1 and Islet1 markers have shown that there was a suppression of HC numbers in the Egr1-morphant retinas at 72 hpf, while only Islet1+ HCs were suppressed at 120 hpf (Figure 6).

*Ptf1a* is a TF that is transiently expressed in AC and HC precursors between 35 and 40 hpf in the INL and is responsible for the commitment of both ACs and HCs in zebrafish [31], frog [36], mouse [34], [37] and chick [38]. In this study, there was an abnormal expression of *ptf1a* in the Egr1-morphant retinas, in which there was an ectopic expression in the central retina at 72 hpf; while the expression of *ptf1a* in the control retinas was restricted to the proliferative MZ (Figure 8C and D). Since *ptf1a* also became restricted to the MZ in the Egr1 morphants by 120 hpf, this observation suggests that Egr1 knockdown led to a prolonged expression of *ptf1a* in the developing neural retina. Hence, it is reasonable to speculate that the reduction in the differentiated ACs and HCs in the Egr1-morphant retinas was caused by a prolonged expression of *ptf1a*. Nonetheless, it should be noted that in the aforementioned earlier studies, the overexpression of *ptf1a* led to an increase in the number of ACs and HCs and vice versa [34], [36], [37], [38], while these phenomena were not observed in the current study. Since the Egr1 level was presumably not perturbed in these earlier studies, the combinatorial effect of Egr1 and Ptf1a may be critical for determining outcome of the cell-type specification. Thus, the results from the current study have indicated that a prolonged expression of *ptf1a* with *egr1* deficiency might lead to a suppression of Parv+ and GABA+ ACs as well as Islet1+ HCs differentiation. Alternatively, the prolonged expression of *ptf1a* was caused by the developmental delay and did not play a role in the suppression of these cell types. In this case, the specific suppression of these ACs and HCs was exclusively caused by the Egr1 knockdown.

The fate of the suppressed ACs and HCs is not currently clear. In the case of ACs, there was a slight reduction of 5E11, a pan-specific AC marker (Figure 4). Thus, the suppression of Parv+ and GABA+ ACs in the Egr1-morphant retinas may simply indicate that these cell types were not formed. It is possible that the precursors of these ACs died, stalled or assumed alterative fate in differentiation. In the case of HCs, the current results indicate that only the number of Islet1+ HCs but not Prox1+ HCs was reduced in the Egr1-morphant retinas (Figure 6). Since Prox1 is a pan-HC marker [31], the lack of a general reduction in the HC number indicates that the Islet1+ HCs might become other HC subtypes in the Egr1-morphant retinas. These possibilities can potentially be determined by knocking down Egr1 in the *Tg(ptf1a:EGFP*) transgenic fish that can label all ACs [39] and HCs [31] and tracing the developmental fate of these cell types in their retinas.

The normal differentiation of various retinal cells is essential for their normal extension of neuronal projections into the plexiform layers. The delay of their differentiation in the Egr1-morphant retinas has contributed to the observed defects in retinal lamination at 72 hpf (Figure 3A & B). Once many of these retinal cells differentiated at 120 hpf (Figures 4 – 7), the retinal lamination issue was substantially improved (Figure 3C & D). Nonetheless, the specific differentiation problems of AC subtypes induced by Egr1 knockdown at 120 hpf (Figure 4) still likely caused a thinner IPL at this stage (Figure 3 & Figure 5).

It has been demonstrated that ACs might play a major role in the early establishment of IPL. For example, the first ACs extended neuronal projections and formed a laminated IPL in normal zebrafish retinas at around 42 hpf [39] and in zebrafish *atoh7*-mutant retinas that lack GCs [40]. In the latter mutant retina, the retinal lamination including the formation of IPL appeared largely normal. The same phenomenon was also observed in the mouse *atoh7*-mutant retinas [41]. BCs and MCs, on the other hand, are not born early enough to mediate the IPL formation. Thus, the identification of *egr1*’s specific role in the development of AC subtypes by the current investigation may facilitate the study of the role of ACs in early IPL formation in the future.

A few *smarca4*-regulated genes, including *p35/cdk5* [12] and *irx7* [14], have been reported to control retinal differentiation and lamination. The current study have provided evidences that *egr1* may also play a similar role in this process. Since it has been reported that *p35* is a downstream effector of *egr1*-regulated neurite outgrowth *in vitro* [42], and that the retinal lamination phenotypes of the Irx7 morphants share a number of similarities with the Egr1 morphants, *egr1* may functionally interact with *p35* and *irx7.* It is expected that our ongoing investigation on their functional relationship will further our understanding of retinal differentiation and lamination.

## Materials and Methods

### Zebrafish maintenance and embryo collection

Zebrafish AB line was maintained according to standard procedures [43]. Parental fish were bred for 15 minutes before embryo collection to ensure all embryos would be collected at a similar stage. Then, embryos were collected, raised at 28°C and staged as described [44]. For *in situ* hybridization, embryos were also treated with 0.003% PTU (Sigma) in E3 medium [45] between 12 and 23 hpf to prevent melanization. All protocols were approved by the Purdue Animal Care and Use Committee.

### Morpholino (MO) injection

To knockdown Egr1 (NCBI Reference Sequence: NM_131248.1), either 3 ng of a translation-blocking MO (*egr1*tMO, sequence: AGCCATCTCTCTGGAGTGTGCTCGG) or 4 ng of a splice-blocking MO (*egr1*sMO, sequence: AAGAGGGATTTAGTGCTTACCTCCA) was injected into the yolk of embryos at one-cell stage as described [45]. Three nanograms of a standard control MO (stdCTLMO, sequence: CCTCTTACCTCAGTTACAATTTATA) was used as the control for *egr1*tMO, and 4 ng of a 5-base mismatch control MO (5misCTLMO, sequence: AACACGGATATAGTCCTTAGCTCCA) was used as the control for *egr1*sMO. All MOs were purchased from Gene Tools or Thermo Scientific (formerly Open Biosystems).

### Quantitative PCR (qPCR)

Total RNAs were extracted from 10 whole embryos at 48, 72, 96 and 120 hpf and reverse transcribed as described [46]. qPCR was performed using SYBR Green PCR Master Mix (Applied Biosystems) and run on an Applied Biosystems 7300 Real-Time PCR System as described [28]. Primers were designed and purchased from Integrated DNA Technologies (IDT). The mature spliced mRNA was amplified by *egr1*-F: 5’- AGTTTGATCACCTTGCTGGAG-3’ (located in exon 1) and *egr1*-R: 5’- AACGGCCTGTGTAAGATATGG-3’ (located in exon 2). *β-actin* was utilized as an internal control, and the primers for its amplification were (β-act-F: 5’-TGCTGTTTTCCCCTCCATTG-3’ and β-act-R: 5’-GTCCCATGCCAACCATCACT-3’).

### *In situ* hybridization

*In situ* hybridization was conducted as described [13]. The riboprobes that were used in this study are as follows: *early growth response 1 (egr1); cone-rod homeobox* (*crx*); *neurogenic differentiation* (*neurod*); *nuclear receptor subfamily 2 group E member 3* (*nr2e3*); *pancreas specific transcription factor 1a* (*ptf1a*); *opsin 1 (cone pigments)*, *short-wave-sensitive 1 (opn1sw1)*; *opsin 1 (cone pigments)*, *short-wave-sensitive 2* (*opn1sw2*); *opsin 1 (cone pigments), long-wave-sensitive 1* (*opn1lw1*) and *rhodopsin* (*rho*).

### Immunohistochemistry

All embryos were collected, fixed and stored according to a standard protocol [14], except for embryos used for GABA immunofluorescence, which were fixed in 4% paraformaldehyde (PFA) plus 0.1% glutaraldehyde. Ten-micrometer-thick transverse cryosections were collected and immunostaining conducted as described [14]. The antibodies used in this study and their dilutions are as follows: mouse anti-zn8 (1:500, ZIRC), mouse anti-Islet1 (1∶50, Developmental Studies Hybridoma Bank), mouse anti-parvalbumin (1∶500, Sigma P3088), rabbit anti-GABA (1∶500, Millipore AB131), mouse anti-5E11 (1∶10, [29]). mouse anti-Prox1 (1∶200, Millipore MAB5652), mouse anti-zpr1 (1∶200, ZIRC), mouse anti-zpr3 (1∶200, ZIRC), Alexa Fluor 488/555 goat anti-rabbit/mouse IgG (1∶1000, Invitrogen). Alexa Fluor 633 phalloidin (1∶50, Invitrogen) was included in the first antibody mixture to stain for F-actin, which would highlight the plexiform layers. 100 ng/mL DAPI was used to counter stain cell nuclei.

### Image acquisition and data analysis

Bright-field and fluorescent images were acquired by a SPOT-RT3™ colour slider camera (Diagnostic Instruments) mounted on an Olympus BX51 fluorescence compound microscope or SZX16 stereomicroscope. Features of the samples in the images were extracted by i-Solution (IMT i-Solution). For GCs, ACs and HCs immunostaining results, their cell counts were normalized by the corresponding retinal areas excluding the optic nerve region. For Islet1+ ACs counting, a line was drawn across the central IPL stained by phalloidin; then, the Islet1+ cells on the INL side were counted. It should be noted that if an Islet+ GC was substantially delaminated, it would be counted as a positive cell by this approach at 72 hpf. Nonetheless, the lack of a difference of Islet1+ ACs between the Egr1 morphant and controls at 120 hpf when the morphants formed a distinctive IPL (Figure 4P) indicates that the number of Islet1+ ACs was not affected by the knockdown at 120 hpf.

### Statistical analysis and data visualization

All standard descriptive statistics and data analyses were performed in SPSS 16.0. The analysis of data for two groups was conducted by Mann-Whitney test, except for IPL thickness analysis, which was conducted by two-tailed Student’s *t-*test. qPCR data were analyzed by the ΔΔCt method [47]. Standard error propagation was used to combine measurement errors of the variables. The qPCR results were reported in ratio of mature mRNA amount in the Egr1 morphants to that in the controls (2∧-ddCt) and the corresponding range in 2∧(-(ddCt ± ddCtErr)). The results are also plotted in Figure 2G. An alpha level of 0.05 was used for all statistical tests.

## Supporting Information

Figure S1 **Amacrine cells immunolabeled by Parv and GABA markers.** (Top) An overlay image of GABA+ (green) and Parv+ (red) cells in a normal WT retina at 72 hpf. (Bottom) A magnified view of the white box at the top. From left to right: GABA, Parv and the overlay image. Many of the Parv+ AC cell bodies were also GABA+ (white arrows), suggesting they might be a subset of GABAergic ACs. Note that there were overlapping and non-overlapping GABA+ and Parv+ regions in the IPL, suggesting that these ACs projected to different sub-laminae in the IPL. Scale bar  =  50 µm for the top image and 10 µm for the bottom images.
(TIF)

Figure S2 ***In situ*** **hybridization of** ***opsins*** **at 72 hpf.** *In situ* hybridization of *opn1lw1 (red;* A & B*), opn1sw2 (blue;* C & D*), opn1sw1 (uv;* E & F) and *rhodopsin (rho;* G & H*)* was conducted with the controls (5misCTLMO) and Egr1 morphants (egr1sMO) collected at 72 hpf. The staining of four *opsins* were strongly detected in the whole ONL of the control retinas (A, C, E and G), while their signal in the Egr1 morphants was restricted to the ventral patch and/or a few ONL cells (arrows in B, D, F and H). The ventral view of the embryos is shown in all pictures. To quantify the signal intensity of *in situ* hybridization, the number of embryos with a specific level of staining (Type 1 - ventral patch staining only, Type 2 – ventral patch staining plus some central PR layer staining, and Type 3 – ventral patch plus full PR layer staining) was counted and analyzed by Mann-Whitney test [14]. The results show that there was a difference in the staining type between the controls and Egr1 morphants for all four *opsins* ([*red opsin*]: control counts (type 1–3): 0, 0, 12; Egr1-morphant counts: 5, 13, 0; U  =  0, *p*-value < 0.001; [*blue opsin*]: control counts: 0, 0, 12; Egr1-morphant counts: 11, 7, 0; U  =  0, *p*-value < 0.001; [*uv opsin*]: control counts: 0, 0, 12; Egr1-morphant counts: 13, 5, 1; U  =  6, *p*-value < 0.001; [*rho*]: control counts: 0, 0, 9; Egr1-morphant counts: 15, 5, 0; U  =  0, *p*-value < 0.001). In this figure, all controls are staining Type 3 while all Egr1 morphants are staining Type 2. Note that the effect of Egr1 knockdown on PR differentiation is likely caused by a delay in development, as the immunostaining of PR markers at 120 hpf shows that the differentiation of PRs in the Egr1 morphants was comparable to the controls (Figure 7). Scale bar  =  100 µm.
(TIF)

Figure S3 ***In situ*** **hybridization of** ***nr2e3, neurod*** **and** ***crx*** **at 72 hpf.** (A & B) The staining of *nr2e3* in the Egr1-morphant retinas was higher from the ventral (B) and dorsal (B’’) views compared with the controls (A & A’’). From the medial view, the PRs that were stained as individual dots were widely distributed in the Egr1-morphant retinas (B’), while they were relatively sparse in the control retinas, especially in the central region (A’). For *neurod* and *crx,* their expression patterns and levels were comparable between the control (C & E) and Egr1-morphant (E & F) retinas. Thus, these observations suggest that *egr1* negatively regulates *nr2e3* but not *neurod* and *crx* at 72 hpf. Nonetheless, since PRs ultimately differentiated relatively normally in the Egr1 morphants at 120 hpf (Figure 7), the results are more consistent with the possibility that the development of PRs was delayed in the morphants. Scale bar  =  100 µm.
(TIF)
