# Supplementary figures and images for: The Role of egr1 in Early Zebrafish Retinogenesis
Source: PLoS One. 2013 Feb 6;8(2):e56108. doi: 10.1371/journal.pone.0056108 (PMC3566060; doi:10.1371/journal.pone.0056108)

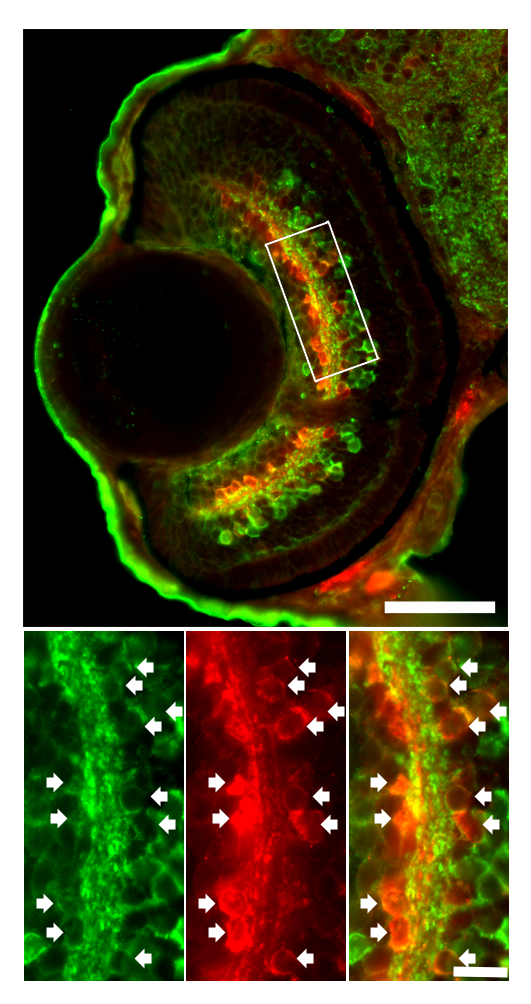

Supplement: Figure S1 — Amacrine cells immunolabeled by Parv and GABA markers. (Top) An overlay image of GABA+ (green) and Parv+ (red) cells in a normal WT retina at 72 hpf. (Bottom) A magnified view of the white box at the top. From left to right: GABA, Parv and the overlay image. Many of the Parv+ AC cell bodies were also GABA+ (white arrows), suggesting they might be a subset of GABAergic ACs. Note that there were overlapping and non-overlapping GABA+ and Parv+ regions in the IPL, suggesting that these ACs projected to different sub-laminae in the IPL. Scale bar = 50 µm for the top image and 10 µm for the bottom images. (TIF) [file pone.0056108.s001.tif]

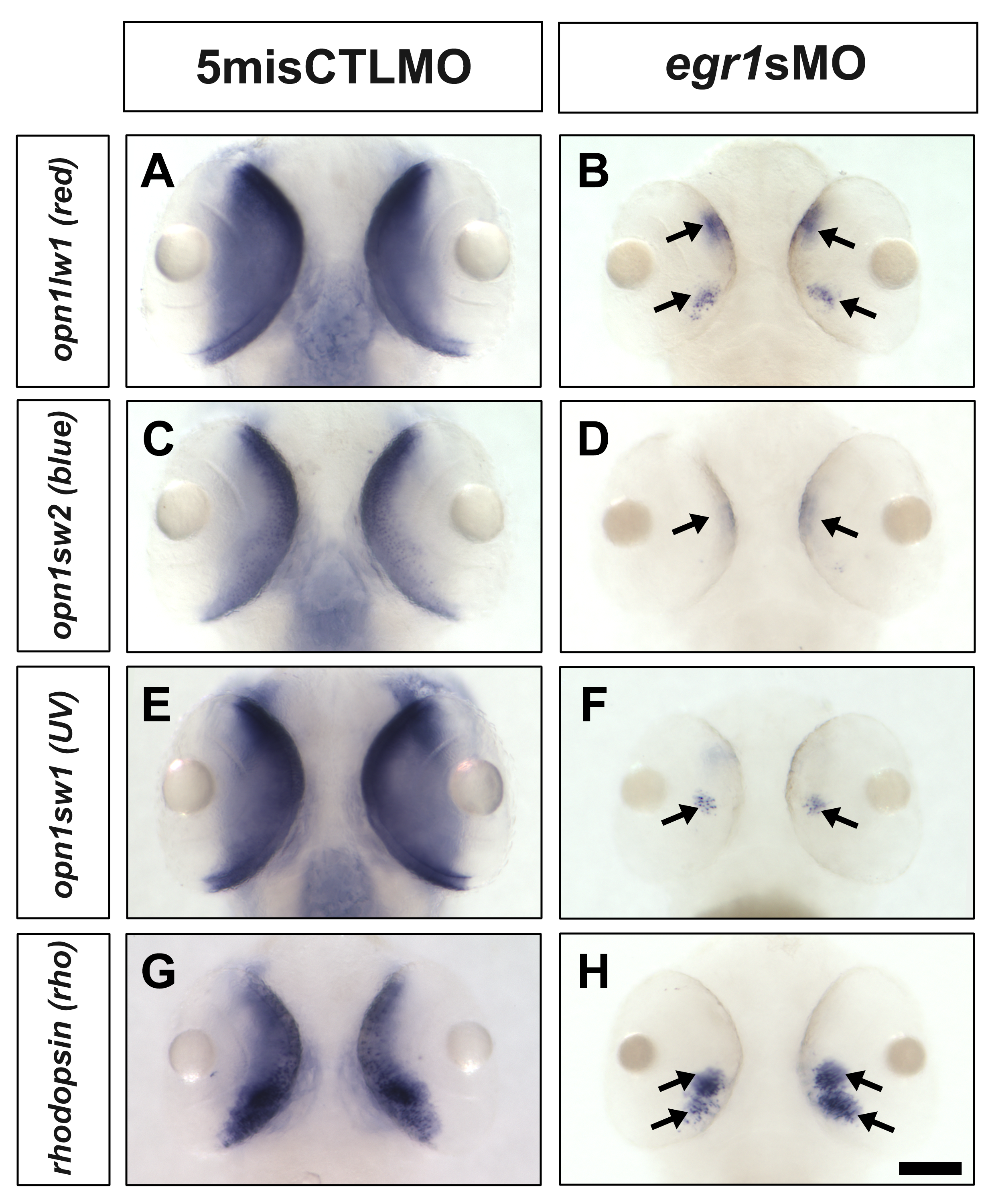

Supplement: Figure S2 — In situ hybridization of opsins at 72 hpf. In situ hybridization of opn1lw1 (red; A & B), opn1sw2 (blue; C & D), opn1sw1 (uv; E & F) and rhodopsin (rho; G & H) was conducted with the controls (5misCTLMO) and Egr1 morphants (egr1sMO) collected at 72 hpf. The staining of four opsins were strongly detected in the whole ONL of the control retinas (A, C, E and G), while their signal in the Egr1 morphants was restricted to the ventral patch and/or a few ONL cells (arrows in B, D, F and H). The ventral view of the embryos is shown in all pictures. To quantify the signal intensity of in situ hybridization, the number of embryos with a specific level of staining (Type 1 - ventral patch staining only, Type 2 – ventral patch staining plus some central PR layer staining, and Type 3 – ventral patch plus full PR layer staining) was counted and analyzed by Mann-Whitney test [14]. The results show that there was a difference in the staining type between the controls and Egr1 morphants for all four opsins ([red opsin]: control counts (type 1–3): 0, 0, 12; Egr1-morphant counts: 5, 13, 0; U = 0, p-value < 0.001; [blue opsin]: control counts: 0, 0, 12; Egr1-morphant counts: 11, 7, 0; U = 0, p-value < 0.001; [uv opsin]: control counts: 0, 0, 12; Egr1-morphant counts: 13, 5, 1; U = 6, p-value < 0.001; [rho]: control counts: 0, 0, 9; Egr1-morphant counts: 15, 5, 0; U = 0, p-value < 0.001). In this figure, all controls are staining Type 3 while all Egr1 morphants are staining Type 2. Note that the effect of Egr1 knockdown on PR differentiation is likely caused by a delay in development, as the immunostaining of PR markers at 120 hpf shows that the differentiation of PRs in the Egr1 morphants was comparable to the controls (Figure 7). Scale bar = 100 µm. (TIF) [file pone.0056108.s002.tif]

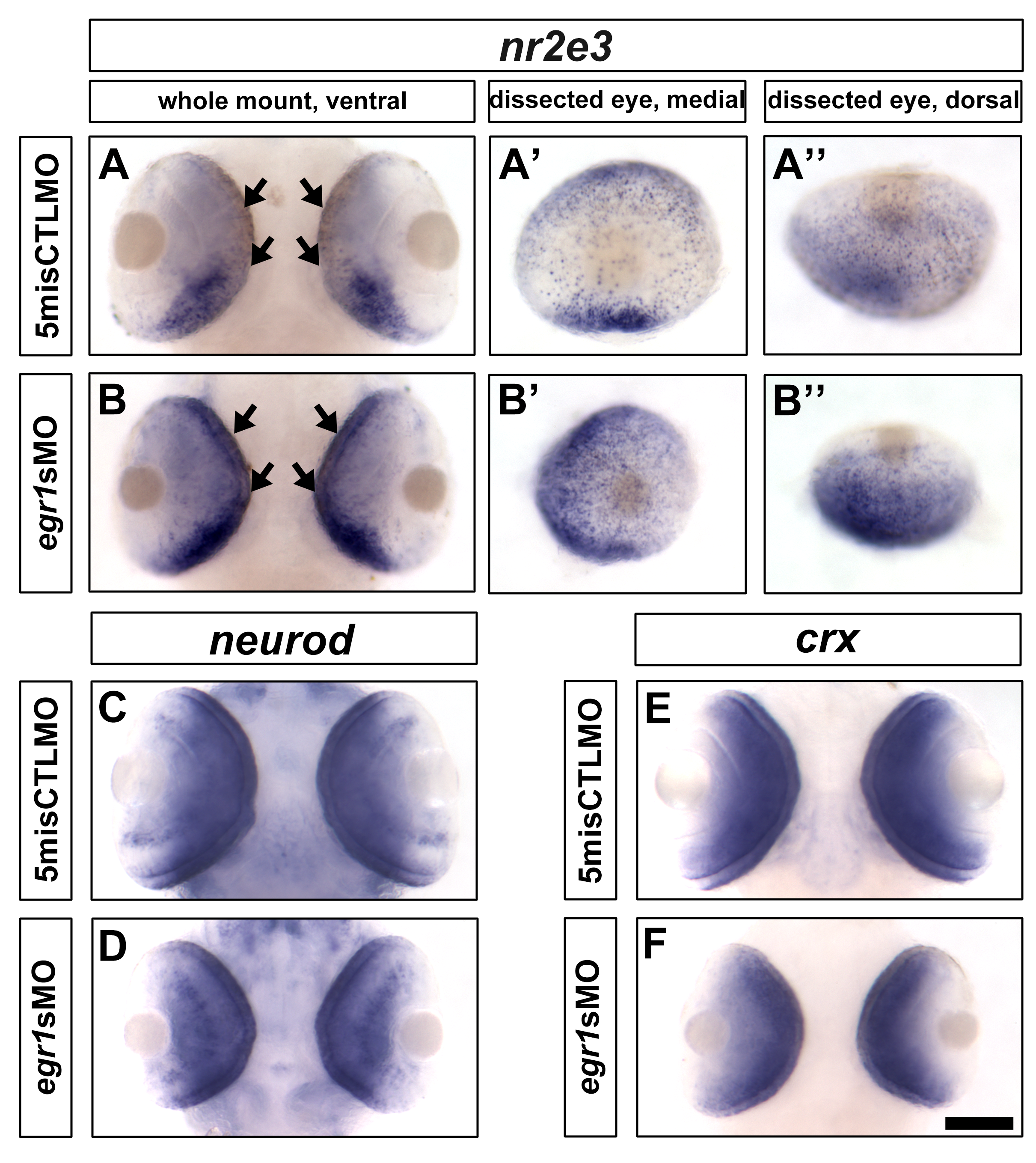

Supplement: Figure S3 — In situ hybridization of nr2e3, neurod and crx at 72 hpf. (A & B) The staining of nr2e3 in the Egr1-morphant retinas was higher from the ventral (B) and dorsal (B’’) views compared with the controls (A & A’’). From the medial view, the PRs that were stained as individual dots were widely distributed in the Egr1-morphant retinas (B’), while they were relatively sparse in the control retinas, especially in the central region (A’). For neurod and crx, their expression patterns and levels were comparable between the control (C & E) and Egr1-morphant (E & F) retinas. Thus, these observations suggest that egr1 negatively regulates nr2e3 but not neurod and crx at 72 hpf. Nonetheless, since PRs ultimately differentiated relatively normally in the Egr1 morphants at 120 hpf (Figure 7), the results are more consistent with the possibility that the development of PRs was delayed in the morphants. Scale bar = 100 µm. (TIF) [file pone.0056108.s003.tif]
